# A Medical Cloud-Based Platform for Respiration Rate Measurement and Hierarchical Classification of Breath Disorders

**DOI:** 10.3390/s140611204

**Published:** 2014-06-24

**Authors:** Atena Roshan Fekr, Majid Janidarmian, Katarzyna Radecka, Zeljko Zilic

**Affiliations:** Department of Electrical and Computer Engineering, McGill University, 3480 University Street Montreal H3A 0E9, Canada; E-Mails: majid.janidarmian@mail.mcgill.ca (M.J.); katarzyna.radecka@mcgill.ca (K.R.); zeljko.zilic@mcgill.ca (Z.Z.)

**Keywords:** respiration rate, breath analysis, accelerometer sensor, Support Vector Machine, breath disorder

## Abstract

The measurement of human respiratory signals is crucial in cyberbiological systems. A disordered breathing pattern can be the first symptom of different physiological, mechanical, or psychological dysfunctions. Therefore, a real-time monitoring of the respiration patterns, as well as respiration rate is a critical need in medical applications. There are several methods for respiration rate measurement. However, despite their accuracy, these methods are expensive and could not be integrated in a body sensor network. In this work, we present a real-time cloud-based platform for both monitoring the respiration rate and breath pattern classification, remotely. The proposed system is designed particularly for patients with breathing problems (e.g., respiratory complications after surgery) or sleep disorders. Our system includes calibrated accelerometer sensor, Bluetooth Low Energy (BLE) and cloud-computing model. We also suggest a procedure to improve the accuracy of respiration rate for patients at rest positions. The overall error in the respiration rate calculation is obtained 0.53% considering SPR-BTA spirometer as the reference. Five types of respiration disorders, Bradapnea, Tachypnea, Cheyn-stokes, Kaussmal, and Biot's breathing are classified based on hierarchical Support Vector Machine (SVM) with seven different features. We have evaluated the performance of the proposed classification while it is individualized to every subject (case 1) as well as considering all subjects (case 2). Since the selection of kernel function is a key factor to decide SVM's performance, in this paper three different kernel functions are evaluated. The experiments are conducted with 11 subjects and the average accuracy of 94.52% for case 1 and the accuracy of 81.29% for case 2 are achieved based on Radial Basis Function (RBF). Finally, a performance evaluation has been done for normal and impaired subjects considering sensitivity, specificity and G-mean parameters of different kernel functions.

## 1. Introduction

Different studies show the importance of monitoring and analyzing the respiration signals in fields such as medicine and physiology [1–4]. Today about 7% of the population of developed countries suffer from Chronic Obstructive Pulmonary Disease (COPD), and it is a growing problem in developing countries. For example, an estimated of 3.7 million people live with COPD in UK, predicted to increase by one-third by 2030, costing £1.2 billion/y [3]. Moreover, professionals in breathing and sleep centers are demanded to assist people with shortness of breath, cardiovascular problems, such as hypertension, atherosclerosis, stroke, heart failure, cardiac arrhythmias, and sudden infant death syndrome (SISD). Therefore, a real-time monitoring of the respiration rhythm plays an important role in both diagnosis and treatment of different disorders. Remote monitoring also helps in prevention and early diagnosis of adult diseases, such as obesity, diabetic ketoacidosis (DKA), brain disorders as well as abnormal breathing of newborns at home. There are different conventional methods for respiration rate measurement including spirometer, body volume changes, nasal thermocouples, impedance plethysmography, inductance pneumography, strain gauge measurements of thoracic circumference, whole-body plethysmography [4], pneumatic respiration transducers, the fiber-optic sensor method [5], the Doppler radar [6], and electrocardiogram (ECG)-based derived respiration measurements [7–9]. However, in spite of their accuracy, these methods are expensive and inflexible, which may bring discomfort to the patients and physicians.

One recent development is the use of motion sensors to detect the small movements of the chest wall that occur during expansion and contraction of the lungs. In preliminary trials on hospital patients, it has been shown that with proper signal processing; this approach can produce results that match closely the measurements of nasal cannula pressure [10]. In [11] an accelerometer and pressure sensors are mounted on the body to obtain the respiratory rate. In this work the data was collected by the data acquisition card, and then the processing has been done in Labview software. A validation of respiratory signal derived from suprasternal, notch acceleration has been investigated by [12] for different body positions. In this paper, the spirometry and strain gauge respirometers (SGR) signals were filtered through an 8th order Butterworth bandpass filter with cut-off frequencies 0.1 Hz and 1 Hz. In order to remove noise from accelerometer data, they used an 8th order Butterworth low-pass filter with a cut-off frequency of 1 Hz. The precision of each sample was 8 bits while the sampling rate was 2000 Hz. The authors show that the respiration rate from the accelerometer has 1.55% error with respect to the spirometer. Their data storage and processing is performed on a computer with their custom build LabVIEW Virtual Instrument. In [13] the respiratory component is also extracted from the accelerometer mounted on the suprasternal notch of subjects. The vibrations are recorded with a transducer electronic data sheets (TEDS) lightweight piezoelectric accelerometer. The results demonstrate the feasibility of implementing an accelerometry-based portable device for respiration recording. The data acquisition is done with a compact system and data was stored in a laptop. Recently, [14] proposed a fusion method for accelerometer and gyroscope signals to calculate the respiration rate. They considered two types of exercises and the respiration rate errors are calculated as 4.6% and 9.54% for the treadmill and leg press, respectively.

In addition to obtaining an accurate respiration rate, several methods have been also suggested for identification of respiration disorders, such as sleep apnea. In fact, statistical features of different signals, such as nasal air flow, the thorax and abdomen effort signals, Electroencephalography (EEG), and ECG are mostly used in the detection. In [15] various feature sets are analyzed and a combination of classifiers are used based on the arterial oxygen saturation signal (SpO_2_) and the ECG in order to evaluate sleep quality and apnea detection. The Bagging with REP Tree classifier achieved 79.75% and 85.89% of sensitivity and specificity respectively, while the overall accuracy obtained 84.40%. In [16] the authors applied the wavelet transforms and an artificial neural network (ANN) algorithm to the EEG signal in order to identify sleep apnea episodes. Recently, [17] proposed a method which used Melfrequency cepstral coefficients (MFCC) as features extracted from respiratory sounds. They applied support vector machine classifier to distinguish normal, obstruction pathology airway and parenchymal pathology. They achieve an average classification accuracy of 90.77%.

Indeed, there are several methods for classification of normal and sleep apnea breathing patterns. However, in this paper five different types of respiration disorders either with or without apnea are considered and remotely classified using hierarchical SVM, based on the best extracted features from accelerometer sensors data. SVM introduced by Vapnik [18] in 1995, is a set of related supervised learning methods used for classification, regression and ranking. SVM classifiers are trained by a learning method from optimization theory which makes a learning bias obtained from statistical learning theory [18–20]. Nonlinear problems in SVM are solved by mapping the n-dimensional input space into a high dimensional feature space in which a linear classifier is constructed which acts as a nonlinear classifier in input space [19]. In this work, different types of SVM classifiers are evaluated on five different breathing disorders, and finally the accuracy, sensitivity, specificity and G-mean parameters are calculated for distinguishing of healthy and unhealthy subjects.

The advanced low-cost and energy efficient data transport architectures for body sensor networks allow the doctors and physicians monitor their patient remotely with serious focus and effect on successful results in healthcare [21]. Therefore, in this paper we address a primary advance in this capability through development of a platform. It delivers required accuracy, continuous monitoring at low cost, and cloud computing for breathing disorders classification. Indeed, in each breathing cycle the volume of the thoracic cavity is changed, resulted from the displacements of the rib cage and diaphragm. Hence, we employ an accelerometer sensor mounted on the subject's chest to capture the movement of the rib cage while also providing more comfort compared to other locations, such as suprasternal notch. The previous approaches are primarily based on the use of offline data loggers and on-board signal processing. However, in our system we use cloud computing which can offer significant advantages over traditional methods, including increased on-line accessibility, scalability, automatic failover and fast automated recovery from failures.

**Figure 1.**
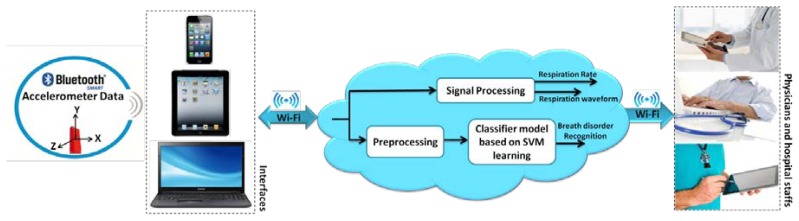
Overall view of the proposed cloud-based respiration monitoring platform.

The accelerometer data is transmitted via Bluetooth Low Energy (BLE) to PC/smartphones and then it is sent to the cloud to be processed and saved, immediately. The algorithms and models run on the backend and feed the application with relevant data in a reasonable amount of time. It is worth mentioning that in case of network disconnection, the data is saved in the intermediate interface. Therefore, the physicians can track the patients wherever they are with devices such as smartphones, tablets or the web regardless of their proximity to the patients. Moreover, in this study the accuracy of respiration rate compared to previous works [14], [12] has been improved in view of rest conditions as well as providing a classification for different breath problems. The overall proposed system is represented in Figure 1. Respiration rate analysis and classification methods are explained in details in the next sections.

In Section 2, a new method is used to accurately calculate the respiration rate. In Section 3, the hierarchical SVM has been applied on respiration signals which derived from the accelerometer to classify different types of the respiration patterns. Experimental results are presented and discussed in Section 4. In Section 5, normal and impaired respirations classification is investigated. Finally, Section 6 concludes the paper.

## 2. Respiration Rate Analysis

**Figure 2.**
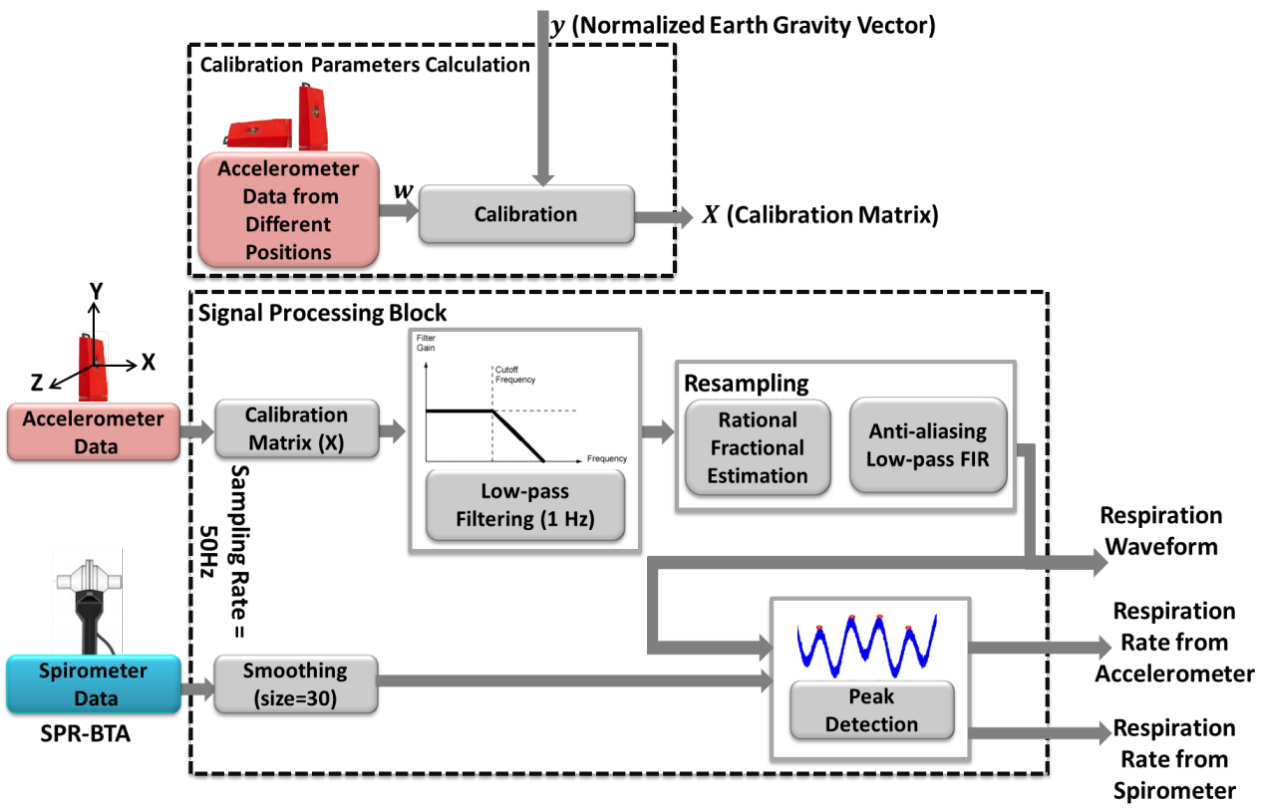
Signal processing of respiration rate analysis.

In this section, we describe a procedure to estimate the respiration rate at rest positions. Figure 2 shows the signal processing methods applied on the accelerometer sensor data to be validated with a reference *i.e.*, SPR-BTA spirometer.

In order to make sure that the sensors' readings are accurate enough to be processed, we calibrate our accelerometer sensor using least square method proposed by [22]. Due to inherent deficiency or aging problems in cyber-biological systems, sensors calibration is suggested. Calibration, which is defined as the process of mapping raw sensor readings into corrected values, can be used to compensate the systematic offset and gain [23]. Generally, calibration of sensors requires experience and special accurate tools; however, a straightforward method to calibrate an accelerometer is performed at 6 stationary positions [22]. We need to collect a few seconds of accelerometer raw data at each position. Then the least square method is applied to obtain the 12 accelerometer calibration parameters. The calibration procedure is simple, and needs to be executed once.

The calibration procedure can be briefly explained as:

(1) $$[\begin{array}{ccc} a_{x^{\prime }} & a_{y^{\prime }} & a_{z^{\prime }} \end{array}]=[\begin{array}{cccc} a_{x} & a_{y} & a_{z} & 1 \end{array}]\cdot \left[\begin{array}{ccc} {\text{acc}}_{11} & {\text{acc}}_{21} & {\text{acc}}_{31}\\ {\text{acc}}_{12} & {\text{acc}}_{22} & {\text{acc}}_{32}\\ {\text{acc}}_{13} & {\text{acc}}_{23} & {\text{acc}}_{33}\\ {\text{acc}}_{10} & {\text{acc}}_{20} & {\text{acc}}_{30} \end{array}\right]$$

(2) y=w.X

where:

- Vector ***w*** is accelerator sensor raw data collected at 6 stationary positions
- Vector ***y*** is the known normalized Earth gravity vector.
- Matrix ***X*** is the 12 calibration parameters that is determined as below:

(3) $$X={\left[w^{T}\cdot w\right]}^{-1}\cdot w^{T}\cdot y$$

**Figure 3.**
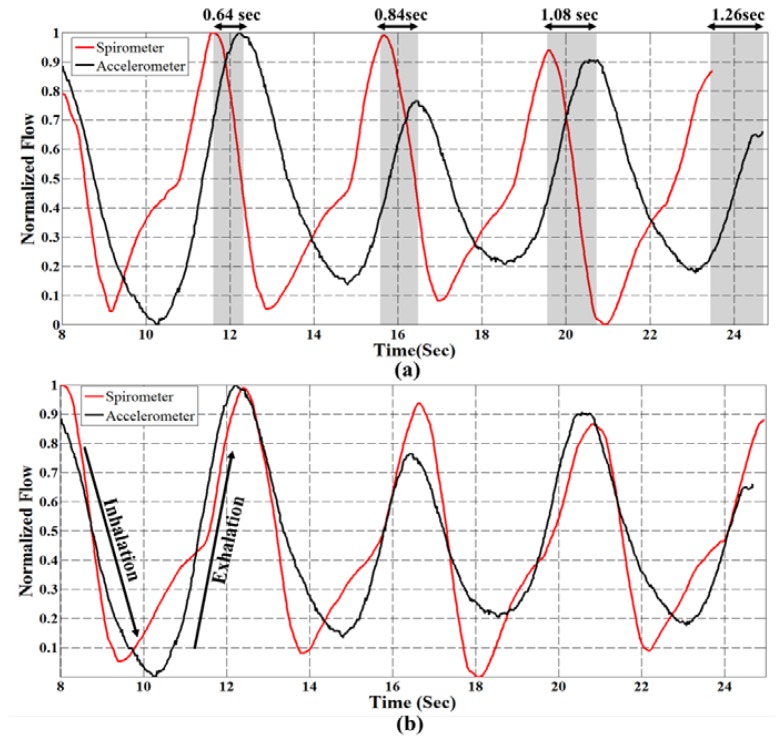
(**a**) The cumulative error of accelerometer and spirometer signals over time before resampling (**b**) Two signals after resampling procedure.

In our experiments, the accelerometer sensor is mounted on the subject's chest for respiration rate analysis. The raw sensor data is filtered through a 10th order Butterworth low pass filter with cut of frequency 1 Hz; and spirometer signal is smoothed with window size 30. Figure 3a, depicts a part of respiration signals from sensor and spirometer for the normal breathing pattern of a 30 years old man. As can be seen, even though two signals seem similar, there is an unwanted cumulative error [14] over time that affected their synchronization. We found out that this type of error on signals occurs when two signals have different sampling frequency. Although, the sampling rates of accelerometer and spirometer are both set to 50 Hz, due to architecture of the inertial measurement unit (IMU), there might be a small difference between the sampling rate and measured frequency. So, to compare the respiration rate, it is essential to ensure that both signals have identical frequencies. For this purpose after rational fraction estimation, we resample our data by an anti-aliasing low pass FIR filter during the resampling process. In our experiments, the sampling rate of the accelerometer sensor was set to 50 Hz, however; the data was logged with about 52.5 Hz (measured frequency). With resampling process explained above, we could compensate the time lead about 0.05 per second (Figure 3). It is worth noting that for resampling process the system automatically checks the number of samples in each analysis window to find the measured frequency of the sensor. Then, to find the best starting point between accelerometer and spirometer, the peak of their cross correlation is obtained while also used for synchronization of two respiration signals. Now, the respiration rate can be computed based on the number of local maxima in the breath signals per minute.

## 3. Respiration Pattern Classification

The main preprocessing steps for breath patterns classifications are calibration, filtering and resampling explained in Section 2. Once the data are processed, they must be collated and labeled based on the different classes such as Normal, Bradapnea, Tachypnea, Cheyn-stokes, Kaussmal, and Biot's respiration patterns. Then a hierarchical structure is built for modeling the classification problem. This is a tree like structure describing the patterns we are trying to classify. At each level of the tree, a SVM classifier is used based on the extracted features and data are separated into one of the branches. Once we reach a leaf node, a final classification is made. The classification structure is shown in Section 4 in more details.

### 3.1. Feature Extraction

Features can be thought of as statistically unique elements of the sensor data, which are used to differentiate diverse classes or states. In the proposed system, classes are the different respiration patterns inferred from various types of breath disorders such as Normal, Bradapnea, Tachypnea, Kaussmal and two types of constraint breathing, such as Cheyn-stokes and Biot's respiration patterns. Features such as energy, mean, maximum, standard deviation, inter-axes correlation and number of local maxima are calculated on both three axes (x,y,z) of the accelerometer and the magnitude of them $\left(\text{Mag}=\sqrt{x^{2}+y^{2}+z^{2}}\right)$. The features are listed in Table 1. The S_i_ corresponding to the signal, and n is number of samples. Besides, we extract the Approximate Entropy (ApEn) from the accelerometer signals as one of the main feature in our classifier explained in the next subsection. Indeed, there are a vast number of diverse features providing freedom in selection that best suit each application. A window of a fixed length (10 s) shifted in increments of 2 s was experimentally selected. We asked the subjects to pause breathing for at least 3 s to simulate the apnea in Cheyn-stokes and Biot's breathing patterns (in our experiments, no subject paused breathing more than about 5 s). The best features are extracted based on the obtained accuracy resulted from the training set.

#### Approximate Entropy (ApEn) Feature

ApEn is a technique introduced by Pincus [24] to quantify the regularity/irregularity of a signal. It has been applied to describe changes in physical activity measures as well as other movement tasks [25]. ApEn has two user-specified parameters: *m*, a positive integer, indicates the length of compared window, and *r* is the tolerance range. It is worth mentioning that, although *m* and *r* are critical in determining the outcome of ApEn, there is no established consensus for choosing these parameters in short data sets, especially for biological data [25]. In our experiments we set *m* and *r* to 5 and 0.15, respectively.

For an *n* sample time series {*u*(*i*): 1 ≤ *i* ≤ *n*}, given *m*, form vector sequences $x_{1}^{m}$ through $x_{N-m+1}^{m}$ as:

(4) $$\begin{array}{cc} x_{i}^{m}=\{u(i),u(i+1),\ldots ,u(i+m-1), & i=1,\ldots ,n-m+1\} \end{array}$$

For each *i* ≤ n – m + 1, let $C_{i}^{m}$ be (n – m + 1)^−1^ times the number of vectors $x_{j}^{m}$within r of $x_{i}^{m}$ which means [26]:

(5) $$C_{i}^{m}(r)={\left(n-m+1\right)}^{-1}(\text{number of j such that d}[x(i)],x(j)\leq r)$$

(6) $$d[x(i),x(j)]=\underset{k=1,2,\ldots ,m}{\max}(\left|u(i+k-1)-u(j+k-1\right|)$$

ApEn is obtained from the Equation (7) as follow [24]:

(7) $$ApEn(m,r)=\underset{n\to \infty }{\lim}[\phi^{m}(r)-\phi^{m+1}(r)]$$

(8) $$\phi^{m}(r)={\left(n-m+1\right)}^{-1}\sum_{i=1}^{n-m+1}\ln C_{r}^{m}(i)$$

The ApEn calculation returns a nonnegative number where higher value shows irregularity of the signal and more regularity resulted from the lower ApEn. Note that, ApEn can be used for small data samples even in real time [24] which makes it suitable for our cloud-based monitoring platform.

### 3.2. Hierarchical Support Vector Machine Classification

**Figure 4.**
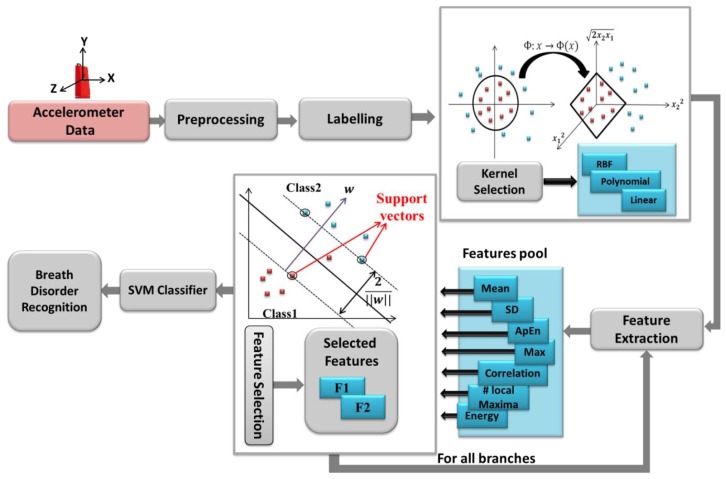
Respiration disorder classification procedure using different kernel functions in hierarchical SVM classifier.

A support vector machine is based on constructing one or a set of hyperplane in a high dimensional space, which can be used for classification purposes. SVM constructs linear functions from a set of labeled training dataset. The linear separator is constructed considering maximum distance from the hyper plane to a fraction of the data points, named support vectors [19] and shown in Figure 4. SVM is designed for binary-classification problems with *n* training samples. Each sample is indicated by (***x****_i_*,*y_i_*) where (i = 1,2,…,n) For a given data set:

(9) $$\{x_{i},y_{i}\},i=1,\ldots ,n,y_{i}\in \{-1,+1\},x_{i}\in R^{d}$$

where *y_i_* is either 1 or −1, indicating the class to which ***x**_i_*, belongs, and ***x**_i_* is a d-dimensional real vector. The notation ***R**^d^* refers to the Cartesian product of *d* copies of ***R***, which is a d-dimensional vector space over the field of the real numbers.

The decision boundary of a linear classifier can be written as follows:

(10) $$w^{T}x+b=0$$

Where ***w*** is a weight vector and *b* is a bias. There are many linear separators; however the SVM aims to obtain the maximum-margin hyperplane from any data point. Indeed, this distance from the decision boundary to the closest data point determines the margin of the classifier. The linear classifier is defined as:

(11) $$f(x)=\text{sign}(w^{T}x+b)$$

Designing linear separator is to find the best *w* and *b* which maximize the geometric margin (2/‖***w***‖), considering *y_i_*(***w****^T^****x****_i_* + *b*) ≥ 1 for all (***x****_i_*,*y_i_*),*i* = 1,…*n*. This can be reformulated as a minimization problem as follow:

(12) $$\begin{array}{c} \underset{w,b}{\min}(\frac{1}{2}{\left\|w\right\|}^{2})\\ s.t.y_{i}(w^{T}x_{i}+b)\geq 1,i=1,\ldots n \end{array}$$

Indeed, this problem is a convex quadratic optimization with quadratic function subject to linear constraints which can transformed into the Lagrangian dual with the Karush-Kuhn-Tucker (KKT) conditions. Lagrange multiplier *α_i_* is linked with every inequality of the linear constrains in the primal problem as follow:

(13) $$\begin{array}{c} \underset{\alpha \geq 0}{\max}\underset{w,b}{\min}\{\frac{1}{2}{\left|\left|w\right|\right|}^{2}-\sum_{i=1}^{n}\alpha_{i}[y_{i}(w^{T}x_{i}+b)-1]\},\\ \begin{array}{cc} \underset{\alpha \geq 0}{\max}\left\{\sum_{i=1}^{n}\alpha_{i}-\frac{1}{2}\sum_{i=1}^{n}\sum_{j=1}^{n}\alpha_{i}\alpha_{j}y_{i}y_{j}x_{i}^{T}x_{j}\right\}, & s.t.\sum_{i=1}^{n}\alpha_{i}y_{i}=0 \end{array} \end{array}$$

Usually only a small parts of *α_i_* are not zero, the corresponding samples are support vectors. Optimal classification function is:

(14) $$f(x)=\text{sign}(w^{T}x+b)=\text{sign}(\sum_{i,j=1}^{n_{sv}}\alpha_{i}y_{i}x_{i}^{T}x_{j}+b)$$

*n_sv_* presents the number of the support vectors. In order to improve linear separability, the original input space is mapped into a high dimensional space which is known as feature space. In this case the decision function of the classifier becomes:

(15) $$f(x)=\text{sign}\left(\sum_{i,j=1}^{n_{sv}}\alpha_{i}y_{i}\phi {\left(x_{i}\right)}^{T}\phi (x_{j})+b\right)\;\text{where}\;\phi :R^{d}\to F$$

A kernel function *K* (***x****_i_*,***x****_j_*) = (x003D5)(***x****_i_*)*^T^*(x003D5)(***x****_j_*) gives the inner product value of ***x****_i_* and ***x****_j_* in the feature space. Therefore the final function becomes:

(16) $$f(x)=\text{sign}\left(\sum_{i,j=1}^{n_{sv}}\alpha_{i}y_{i}K(x_{i},x_{j})+b\right)$$

Three types of kernels are evaluated in this paper which are:

- Linear kernel: *K*(***x**_i_*,***x**_j_*) = ***x**_i_^T^**x**_j_*,
- Polynomial kernel with degree 3: *K*(***x**_i_*,***x**_j_*) = (***x**_i_^T^**x**_j_* + 1)^3^,
- Radial basis function (RBF) kernel: $K(x_{i},x_{j})=\exp(-\frac{{\left|\left|x_{i}-x_{j}\right|\right|}^{2}}{2\sigma^{2}})$

The SVM performance depends on the choice of the kernel function to transform data from input space to a higher dimensional feature space. There are no defined rules for choosing the kernel type, except satisfactory performance by simulation study [19]. Figure 4 depicts the whole system flow of breath disorder recognition architecture.

## 4. Experimental Results and Data Analysis

**Figure 5.**
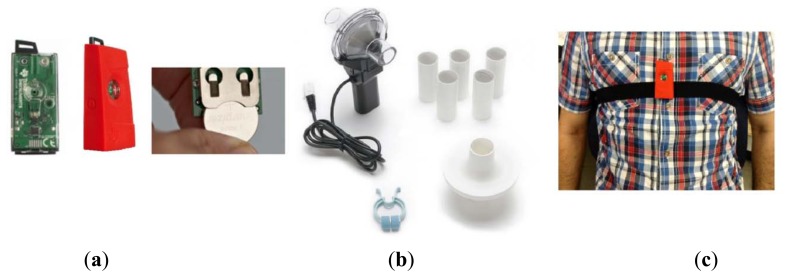
(**a**) Sensory node (**b**) Spirometer (**c**) Hardware module being worn.

In this section, we provide experimental results on 12-bit resolution data derived from SensorTag [27] with three-axis low-power KXTJ9 accelerometer sensor (Figure 5a). The data is transmitted via CC2541 BLE, a new standard that allows Bluetooth equipment to run for long time on a single coin cell battery. It is worth noting that our node is fully radio type approved for US, Europe, Japan and Canada. The received sensors data are stored in the cloud in order to real-time or further analysis. The SPR-BTA spirometer signal is used as our reference measures the oral breathing only (Figure 5b), so a nose clip was used to prevent nasal breathing during recordings. The Go!Link USB sensor interface is used as the data logger of our spirometer.

### 4.1. Test Setup

The participants of this study were five males and six females aged 4 to 48 with Mean ± SD, 26.54 ± 11.9026. They were instructed how to perform each breath exercise before their recording sessions. The experimental trials lasted for about 45 min per subject. We asked the subjects to perform Normal, Bradapnea, Tachypnea, and Cheyn-stokes patterns, each for 2 min (6000 samples) and the other two types for 1 min with a 3 min rest interval. For simulating apnea in Cheyn-stokes and Biot's breathing exercises, we requested the participants to pause breathing for at least 3 s. The sensor was mounted on the subject's chest in the middle of sternum region (Figure 5c) and secured by a soft and elastic strap which is easy to attach and comfortable to wear. In the trial session, the subjects were in the lying position; however, the rest positions or activities in which rib cage is stationary could be considered.

### 4.2. Accelerometer Driven Respiration Validation

In this section, first the correlation between the spirometer and accelerometer signal is calculated on 11 different subjects with various ages, each for five types of breathing disorders. Next, the respiratory rate which recognized as important indicators of physical health or the exacerbation of medical conditions is calculated. In our test, the subjects are asked to perform six breathing patterns *i.e.*, Normal, Bradapnea, Tachypnea, Cheyn-stokes, Kaussmal, and Biot's.

Bradapnea is regular in rhythm but slower than normal in rate. Tachypnea is the condition of rapid breathing, with respiration rate higher than 20 respirations per minute (rpm). Tachypnea may occur due to physiological or pathological problems [28]. Cheyn-stokes breathing pattern determined by gradually increasing, then decreasing the lung volume with a period of apnea. People suffering from central sleep apnea syndrome (CSAS) have the same breathing pattern at sleep [29]. Kussmaul which is defined as a rapid, deep and laboured breathing type usually occurs in diabetics in diabetic ketoacidosis [30].

**Figure 6.**
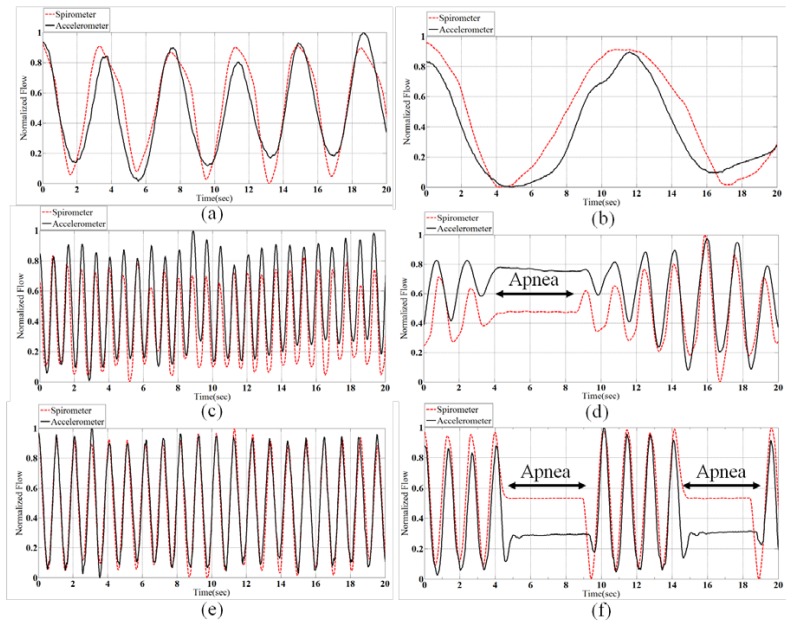
(**a**) Accelerometer driven respiration and spirometer signals for Normal breathing, (**b**) Bradapnea, (**c**) Tachypnea, (**d**) Cheyn-stokes, (**e**) Kussmaul and (**f**) Biot's breathing patterns.

Biot's breathing is characterized by periods of rapid respirations followed by regular periods of apnea. There are different reasons which causes Biot's breathing, such as damage to the medulla oblongata by stroke (CVA) or trauma, or pressure on the medulla due to uncal or tenorial herniation and prolonged opioid abuse [30]. Figure 6 shows samples of all normalized patterns extracted from spirometery and accelerometer.

**Figure 7.**
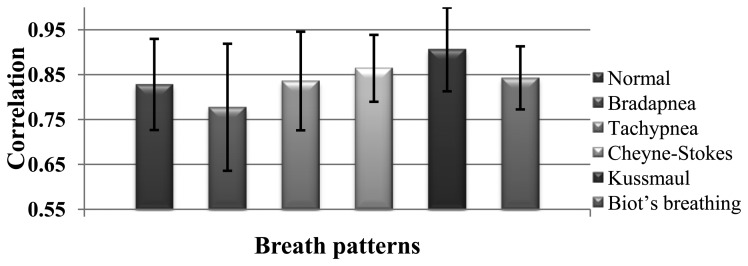
Average correlation between spirometer and accelerometer signals and the standard deviations for five different disorders.

The correlation of spirometer and accelerometer signals of all subjects for each breath pattern is shown in Figure 7. For example, in Normal pattern we have 22-minute data for 11 subjects and the correlation of the accelerometer and spirometer signals is achieved 0.83. According to Figure 7, the mean of the obtained correlations of all breathing patterns is 0.84 which shows a very close correspondence of the sensor and spirometer data.

According to the procedure explained in section 2, the average respiration rates are extracted from the 10-second windows for 11 subjects and 6 breath conditions. The overall error in respiration rate calculation is obtained 0.53% considering SPR-BTA spirometer as the reference. Thus, we could obtain better accuracy for respiration rate compared to [12], dual straingauge respirometers [31], and [14] in lying position using a single accelerometer. The details of the experiments are brought in Table 2. The average volume of air that was inhaled/exhaled per breath for each subject is also listed in Table 3. It confirms that the subjects were not over emphasizing the breathing movements. For instance, it can be seen that standard deviation in Cheyn-stokes breath is more than other types, meaning that subjects were correctly changed the air volumes in this exercise.

### 4.3. Results and Performance Evaluation of Hierarchical SVM Classification

**Figure 8.**
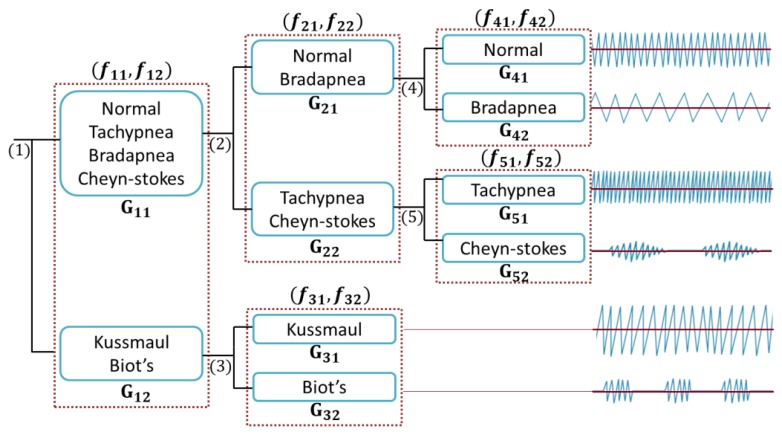
Tree structure of proposed hierarchical SVM classifier.

The proposed classification method is performed by using a cloud-computing and the results are sent to our monitoring platform. We categorized breathing data into six classes based on the features evaluations. Considering the proposed tree structure in Figure 8, first, the data are automatically separated into G11 and G12 groups, employing the assigned labels in “Labelling” section (see Figure 4) and then are passed in to the second and third levels.

We have evaluated the performance of the proposed classification while it is individualized to every subject (case 1) as well as considering all subjects (case 2). Five samples of extracted features and accuracies in training phase for an individual subject are listed in Table 4 and the highlighted row corresponds to the best selected features. Table 5 also provides the best extracted features in terms of training accuracy while the system is trained with all subjects' data. The accuracies in both tables are achieved while the training set is the same as validation set. All simulations were carried out using the Radial Basis kernel Function (RBF) with σ = 1 and A in Tables 4 and 5 denotes the training accuracy calculated by our SVM classifier for each branch. We used linear and non-linear SVM classifiers on each level of our tree structure while training the system with randomly chosen 70% of the data. The performance evaluation is done by testing the remaining 30% of the data.

**Figure 9.**
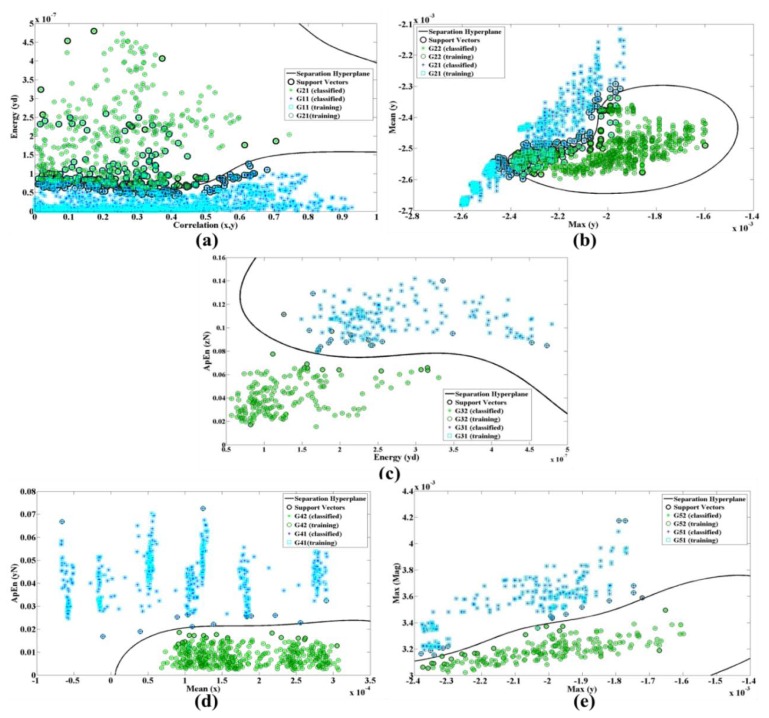
Selected features for (**a**) The first branch (**b**) Second branch, (**c**) Third branch, (**d**) Forth branch (**e**) and fifth branch of our classification structure with the nonlinear trend of Radial Basis Function kernel function considering all subjects' data.

In Figure 8, G_ij_ represents the j_th_ group and f_ik_ is the k_th_ feature of the i_th_ branch. Figure 9a shows the energy along axes y of the accelerometer *versus* the correlation of dimension x and y.

The ApEn is applied on the normalized data while energy performs on x, y and z dimension after removing DC levels. Figure 9 shows the nonlinear trend plots for the different features in all branches of our classification structure while only a single system was trained with all subjects' signals (case 2).

There are totally 168 and 42 training trials for G_11_ and G_12_ for training subject's data individually. The numbers of trials for training all subjects' data are 1680 and 378, respectively. We calculate all combinations of defined features in each branch and then select the one with the best SVM training accuracy.

The processing window for testing data was experimentally selected to be 10 s in order to include at least one complete cycle including pause period (Apnea) for Cheyn-stokes and Biot's breathing patterns. As we mentioned before, the window is shifted in increments of 2 s. The average classification accuracy of 94.50% was obtained for RBF kernel function for 11 subjects while the proposed classification is individualized to every subject. In this case, the system is effectively tuned to every individual and the subjects can either perform training at home or during periodic visits to breath specialist or a physician. The classification performance of 81.29% was also attained when only a single system was trained with all subjects' data.

**Figure 10.**
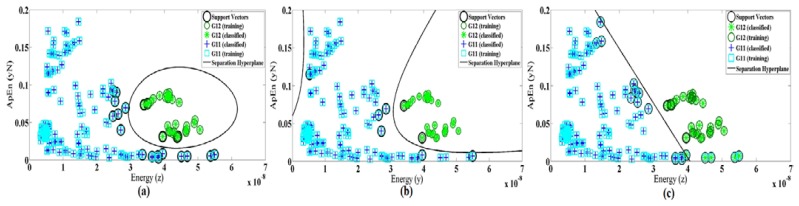
SVM classification based on (**a**) RBF (**b**) Linear, and (**c**) Polynomial degree 3 kernel functions for an average of magnitude signal *vs*. standard deviation of Y axes.

As performance of SVM depends on the choice of the kernel function, we also evaluate the linear and polynomial degree 3 of kernel functions. Figure 10 demonstrates the differences of kernel function for features Max(z) and SD(y) as an example. Table 6 shows the selected features and accuracy for three types of kernel function in both cases 1 and 2. It also shows that the best training accuracy for each classifier obtained with different feature set. The classification of the dataset using the selected features in case 1 gives an average test accuracy of 94.52%, 93.15% and 84.93% for RBF, polynomial and linear kernel functions, respectively. In case 2, the test accuracy of the single system is 81.29%, 78.12% and 69.95% for RBF, polynomial and linear kernel functions, respectively. Therefore, we conclude that for our system (in both cases) the radial basis function results in better accuracy rather than the other two methods.

### 4.4. Normal and Impaired Respirations Classification

In another aspect, our classification problem could be modeled as a binary classification for detecting healthy (positive) or unhealthy (negative) subjects. In this case, *TP*, *FP*, *FN*, *TN* stand for true positives, false positives, false negatives and true negatives correspondingly. The performance of the classifier was quantified based on its sensitivity, specificity and the overall accuracy. It is worth mentioning that sensitivity is also called positive class accuracy or true positive rate, while specificity called negative class accuracy or true negative rate.

Another parameter often used is the geometric mean of sensitivity and specificity (G-mean) which is defined as the square root of the product between sensitivity and specificity as Equation (18). The average values of sensitivity, specificity and geometric mean of SVM classifications for 11 subjects with different kernel functions are shown in Table 7. Table 8 also shows the classification performance of our single model. The values of TP, FP, FN, TN are presented according to the logged data of Normal and impaired breaths, e.g., there are 18 and 72 testing trials for each subject's Normal and impaired breathing patterns, respectively

(17) $$\begin{array}{ccc} Sensitivity=\frac{TP}{TP+FN}, & Specificity=\frac{TN}{TN+FP}, & Accuracy=\frac{TP+TN}{TP+FN+FP+TN} \end{array}$$

(18) $$G-mean=\sqrt{Sensitivity\times Specificity}$$

Therefore, the best classification parameters are achieved for distinguishing between Normal and impaired respiration patterns while using RBF kernel function.

## 5. Conclusions

In this paper, we presented an accurate, reliable and real-time respiration monitoring system. In fact, early identification through such portable monitoring systems and timely treatment of exacerbations can decrease the hospital admissions and slow deterioration while reducing early mortality and disease costs [32]. We presented a procedure to obtain the respiration rate with negligible error *i.e.*, 0.53% for rest positions. The results reveal the potential of remote accelerometer based respiration monitoring as a simple, convenient and low-cost method. Besides, a hierarchical SVM has been proposed to classify five types of respiratory problems for the purposes of diagnosis. Three different kernel functions are evaluated and finally, the radial basis function (RBF) was shown to outperform the classification accuracy of linear and polynomial functions. We also tested our method for normal and impaired breathing classification and the comparison demonstrated that RBF kernel function is again better in terms of accuracy, specificity, sensitivity and G-mean. The results permit to consider SVM analysis as a promising methodology to study the respiratory patterns in patients with breath disorders. Utilizing cloud computing which provides vast pool of computation power and unlimited storage space let the physicians share information together and precisely diagnosis the breathing diseases. As a future work, we are going to add respiratory therapy in the platform is such a way that professional experts can remotely monitor the patient's progressing in performing the prescribed breathing exercises.

## Figures and Tables

**Table 1.** The features for SVM classification.

**Features**	**Description**
Mean	$\bar{\text{s}}=\frac{1}{\text{n}}\sum_{\text{i}=1}^{\text{n}}{\text{s}}_{\text{i}}$, where s_i_ corresponds to the samples, i = 1, …, n
Standard Deviation	$\text{SD}=\sqrt{\frac{1}{\text{n}}\sum_{\text{i}=1}^{\text{n}}{{\text{(s}}_{\text{i}}-\bar{\text{s}})}^{2}}$, where s_i_ corresponds to the samples, i = 1, …, n
Inter-axis Correlation	$r_{\vec{s_{1}},\vec{s_{2}}}=\frac{\text{n}({\text{∑}}_{i=1}^{\text{n}}{{\text{s}}_{1}}_{i}{{\text{s}}_{2}}_{i})-({\text{∑}}_{i=1}^{\text{n}}{{\text{s}}_{1}}_{i})({\text{∑}}_{i=1}^{\text{n}}{{\text{s}}_{2}}_{i})}{\sqrt{\left[\text{n}{\text{∑}}_{i=1}^{\text{n}}{{{\text{s}}_{1}}_{i}}^{2}-{\left({\text{∑}}_{i=1}^{\text{n}}{{\text{s}}_{1}}_{i}\right)}^{2}][n{\text{∑}}_{i=1}^{n}{{{\text{s}}_{2}}_{i}}^{2}\text{−}{\left(\sum_{i=1}^{n}{{\text{s}}_{2}}_{i}\right)}^{2}\right]}}$ where ***s***_1i_ and ***s***_2i_ are samples from two axes, i = 1, …, n
Energy	$\text{e=}\sum_{\text{i}=1}^{\text{n}}{\text{s}}_{\text{i}}^{2}$ , where s_i_ corresponding to the samples, i = 1, …, n
Approximate Entropy	$\text{ApEn}(\text{m},\text{r},\text{n})=\frac{1}{\text{n}-\text{m}+1}{\text{∑}}_{\text{i}=1}^{\text{n}-\text{m}+1}\ln C_{\text{r}}^{\text{m}}(i)-\frac{1}{\text{n}-\text{m}}{\text{∑}}_{\text{i}=1}^{\text{n}-\text{m}}\ln C_{\text{r}}^{\text{m}+1}(i)$ , n is the number of samples in time series s_i_, *r* is the tolerance range and *m* is the length of compared window and *C* is the correlation integral [‎26]
Maximum	Max (**s**, w) = the maximum value of **s** in window size w.

**Table 2.** Respiration rate measurements with both accelerometer and spirometer for different subjects.

**Subject ID**	**Gender/Age**	**Respiration Rate with Accelerometer (rpm)**						**Respiration Rate with Spirometer (rpm)**					

		**Normal**	**Bradapnea**	**Tachypnea**	**Cheyn-stokes**	**Kussmaul**	**Biot's**	**Normal**	**Bradapnea**	**Tachypnea**	**Cheyn-stokes**	**Kussmaul**	**Biot's**
**1**	M/48	13.01	7.45	46.73	37.03	17.44	33.70	13.87	7.46	46.58	36.80	17.21	33.79
**2**	F/37	26.66	19.18	104.89	68.80	47.43	38.86	26.52	19.10	104.16	69.44	47.24	39.01
**3**	M/30	15.82	6.34	108.69	61.64	55.14	36.36	15.85	6.39	108.69	61.50	54.64	36.43
**4**	M/29	25.90	8.52	53.09	44.52	69.35	27.80	26.01	8.47	52.79	44.29	69.75	27.66
**5**	F/28	24.27	17.42	30.00	34.96	50.00	76.92	24.23	17.52	29.97	34.88	49.88	77.41
**6**	F/28	23.62	19.82	25.02	36.23	50.00	33.70	23.78	19.60	24.97	36.40	49.83	33.63
**7**	F/28	24.56	9.63	74.62	51.72	51.72	34.70	24.50	9.70	74.25	51.42	51.28	34.66
**8**	F/27	25.90	11.78	53.95	37.50	39.78	45.80	25.64	11.75	53.83	37.40	39.57	45.91
**9**	F/24	16.42	14.45	113.92	26.93	48.61	33.18	16.23	14.63	113.92	26.78	48.83	33.11
**10**	M/9	20.17	NA	89.28	NA	NA	NA	20.54	NA	89.82	NA	NA	NA
**11**	M/4	31.25	28.51	NA	NA	NA	NA	30.98	28.41	NA	NA	NA	NA

Respiration per minute (rpm). Two kids were asked to perform only two breathing patterns out of Normal, Bradapnea and Tachypnea types. NA (Not Applicable) here means that the kids did not perform the trials.

**Table 3.** The average volume of air (liter) inhaled/exhaled per breath for each subject.

**Subjects ID**	**Normal**	**Bradapnea**	**Tachypnea**	**Cheyn-stokes**	**Kussmaul**	**Biot's**
**1**	0.58 ± 0.0036	0.72 ± 0.0249	1.22 ± 9.58E-04	2.78 ± 0.4120	3.20 ± 0.0030	2.78 ± 0.0030
**2**	0.67 ± 0.0088	1.15 ± 9.33E-04	0.53 ± 6.67E-05	1.32 ± 0.2660	2.22 ± 0.0048	2.25 ± 0.0048
**3**	0.54 ± 0.0015	1.54 ± 0.0021	0.53 ± 6.25E-04	0.88 ± 0.3938	1.34 ± 0.0044	1.70 ± 0.0044
**4**	0.64 ± 3.00E-04	3.12 ± 0.0171	0.41 ± 0.0014	1.38 ± 0.1649	2.85 ± 0.0033	3.50 ± 0.0033
**5**	0.80 ± 0.0023	0.93 ± 0.0013	1.51 ± 0.0065	1.27 ± 0.2107	1.83 ± 0.0028	1.36 ± 0.0028
**6**	0.72 ± 0.0016	1.50 ± 0.0017	0.46 ± 1.00E-04	1.00 ± 0.2632	1.22 ± 0.0013	1.03 ± 0.0013
**7**	0.40 ± 2.33E-04	0.71± 0.0016	0.65 ± 4.33E-04	1.02 ± 0.0544	1.31 ± 0.0018	1.59 ± 0.0018
**8**	0.64 ± 0.0025	1.71 ± 0.0305	0.58 ± 0.0028	1.15 ± 0.2871	1.42 ± 2.33E-04	1.78 ± 2.33E-04
**9**	0.59 ± 0.0019	0.79 ± 0.0032	0.83 ± 0.0043	0.93 ± 0.0780	2.39 ± 0.0036	2.51 ± 0.0036
**10**	0.54 ± 0.0018	NA	0.38 ± 9.67E-04	NA	NA	NA
**11**	0.30 ± 4.67E-04	0.27 ± 5.67E-04	NA	NA	NA	NA

**Table 4.** Five samples of all features combinations and training accuracies for different branches of the proposed classification for case 1.

**Branch #1**			**Branch #2**			**Branch #3**			**Branch #4**			**Branch #5**		
*f*_11_	*f*_12_	*A(%)*	*f*_21_	*f*_22_	*A(%)*	*f*_31_	*f*_32_	*A(%)*	*f*_42_	*f*_42_	*A(%)*	*f*_51_	*f*_52_	*A(%)*
SD(x)	ApEn(z_N_)	98	SD(y)	Mean(Mag)	100	SD(y)	SD(x)	100	E(y_d_)	SD(x)	100	SD(y)	P(z)	100
Max(x)	Mean(x)	96	Mean(x)	ApEn(y_N_)	100	SD(y)	P(x)	100	SD(z)	Max(x)	100	Max(z)	P(x)	100
Max(y)	Mean(z)	96	Corr(x, y)	Max(Mag)	100	SD(y)	P(y)	100	SD(y)	Mean(y)	100	Max(x)	ApEn(y_N_)	100
SD(x)	Max(y)	94	SD(y)	Max(y)	98	ApEn(x_N_)	ApEn(y_N_)	100	SD(y)	ApEn(x_N_)	100	SD(y)	Max(y)	97
Max(y)	E(x_d_)	94	SD(y)	SD(y)	97	SD(z)	Mean(x)	95	Mean(x)	P(x)	92	SD(y)	Max(Mag)	94

E(.) is the energy of a signal, Corr(.) refers to the correlation of the signals, Max(.) shows the maximum of the signal, x_d_, y_d_ and z_d_ correspond to x, y and z after removing the DC levels. x_N_, y_N_ and z_N_ are the normalized values of the accelerometer data. P(.) is the number of local maxima derived from corresponding signal.

**Table 5.** The best selected features combinations and training accuracies for different branches of the proposed classification for case 2.

**Branch #1**			**Branch #2**			**Branch #3**			**Branch #4**			**Branch #5**		
*f*_11_	*f*_12_	*A(%)*	*f*_21_	*f*_22_	*A(%)*	*f*_31_	*f*_32_	*A(%)*	*f*_42_	*f*_42_	*A(%)*	*f*_51_	*f*_52_	*A(%)*
Corr(x, y)	E(y_d_)	92	Max(y)	Mean(y)	94	E(y_d_)	ApEn(z_N_)	98	Mean(x)	ApEn(y_N_)	99	Max(y)	Max(Mag)	99

**Table 6.** Selected features and training accuracy for different branches of the proposed classification with different kernel functions for both cases.

**Case 1**	**Branch #1**			**Branch #2**			**Branch #3**			**Branch #4**			**Branch #5**		
Best Features	*f*_11_	*f*_12_	*A(%)*	*f*_21_	*f*_22_	*A(%)*	*f*_31_	*f*_32_	*A(%)*	*f*_42_	*f*_42_	*A(%)*	*f*_51_	*f*_52_	*A(%)*
RBF	SD(x)	ApEn(z_N_)	98	SD(y)	Mean(Mag)	100	SD(y)	SD(x)	100	E(y_d_)	SD(x)	100	SD(y)	P(z)	100
Linear	Max(x)	Mean(x)	93	Max(Mag)	Mean(Mag)	97	P(z)	E(Mag)	100	Max(x)	ApEn(z_N_)	100	SD(y)	P(y)	100
Polynomial	SD(x)	ApEn(x_d_)	97	SD(y)	P(y)	100	SD(y)	SD(x)	100	SD(y)	Max(Mag)	100	SD(z)	SD(y)	100
**Case 2**	**Branch #1**			**Branch #2**			**Branch #3**			**Branch #4**			**Branch #5**		
Best Features	*f*_11_	*f*_12_	*A(%)*	*f*_21_	*f*_22_	*A(%)*	*f*_31_	*f*_32_	*A(%)*	*f*_42_	*f*_42_	*A(%)*	*f*_51_	*f*_52_	*A(%)*
RBF	Corr(x,y)	E(y_d_)	92	Max(y)	Mean(y)	94	E(y_d_)	ApEn(z_N_)	98	Mean(x)	ApEn(y_N_)	99	Max(y)	Max(Mag)	99
Linear	ApEn(z_N_)	SD(x)	81	SD(y)	Mean(Mag)	86	P(z)	E(Mag)	94	E(y_d_)	ApEn(y_N_)	97	Max(y)	P(z)	99
Polynomial	ApEn(z_N_)	P(y)	89	SD(y)	SD(x)	90	P(z)	SD(y)	95	Mean(y)	P(z)	97	SD(z)	SD(y)	99

**Table 7.** Test performances of the classification for case 1 (in average for 11 subjects).

**Evaluation Parameters**	***TP***	***TN***	***FN***	***FP***	**Sensitivity**	**Specificity**	**Accuracy**	**G-mean**
RBF	18	72	0	0	1	1	1	1
Polynomial	18	67.97	0	4.03	1	0.94	0.95	0.97
Linear	16.28	72	0	1.72	1	0.97	0.98	0.98

**Table 8.** Test performances of the classification for case 2.

**Evaluation Parameters**	***TP***	***TN***	***FN***	***FP***	**Sensitivity**	**Specificity**	**Accuracy**	**G-mean**
RBF	167	647	37	31	0.81	0.95	0.92	0.96
Polynomial	161	641	43	37	0.78	0.94	0.91	0.95
Linear	150	626	58	48	0.72	0.93	0.88	0.94
